# Reconstruction of Delayed Proximal Interphalangeal Joint Fracture-Dislocation Using Hemi-Hamate Arthroplasty: A Case Report

**DOI:** 10.7759/cureus.52797

**Published:** 2024-01-23

**Authors:** Rishikesh V Sawant, Nilesh Darawade, Darshankumar Sonawane, Anish Tawde, Nikhil Bhandari

**Affiliations:** 1 Department of Orthopedics, Bharati Vidyapeeth Medical College and Hospital, Pune, IND; 2 Department of Hand Surgery, Shubhamkar Hand Clinic, Pune, IND; 3 Department of Arthroplasty, Krishna Institute of Medical Sciences (KIMS) Sunshine Hospital, Hyderabad, IND; 4 Department of Orthopedics, Maharashtra Institute of Medical Education and Research (MIMER) Medical College, Pune, IND

**Keywords:** fracture dislocation, graft, articular comminution, hemi-hamate, proximal interphalangeal joint

## Abstract

This case report aims to delineate the clinical outcomes and technical considerations of hemi-hamate arthroplasty in the reconstruction of a delayed proximal interphalangeal (PIP) joint fracture-dislocation. It underscores the procedure's viability as a reconstructive option for complex finger injuries with delayed presentation.

A 23-year-old male presented six weeks post-injury with a PIP joint fracture-dislocation of the left index finger. Traditional management options were limited due to the delayed presentation and the nature of the injury. A surgical intervention was performed using an autologous osteochondral hemi-hamate graft to reconstruct the articular surface. Herein, we describe the detailed surgical steps, postoperative care, and rehabilitation protocols.

Over a five-month follow-up period, the patient demonstrated significant functional improvement. The range of motion in the PIP joint increased substantially, with a notable reduction in pain levels. Radiographic assessments showed successful graft incorporation and joint alignment. The patient reported satisfaction with the aesthetic and functional outcome, highlighting an enhanced quality of life post-surgery.

Hemi-hamate arthroplasty emerges as a favorable surgical option for delayed PIP joint fracture-dislocations, offering improved articular congruity, joint stability, and functional outcomes. This case contributes to the growing body of evidence supporting the procedure's effectiveness and underscores the importance of considering innovative approaches in complex hand injuries.

## Introduction

Proximal interphalangeal (PIP) joint injuries are a common yet potentially complex challenge in orthopedic and hand surgery, often resulting from sports injuries, falls, or direct trauma [1]. The PIP joint's critical role in hand function makes timely and effective management essential to prevent long-term disability and preserve hand dexterity.

Traditional management of PIP joint fracture-dislocations involves closed reduction and immobilization [2], external fixation, or open reduction and internal fixation (ORIF) [3]. However, in cases of delayed presentation or when primary treatments fail, reconstructive options become limited and more complex. Hemi-hamate arthroplasty has emerged as a promising technique for reconstructing the articular surface of the PIP joint, particularly in patients with comminuted articular fractures or those presenting late, where other options might lead to suboptimal outcomes [4].

This case report details the use of hemi-hamate arthroplasty in a patient with a delayed presentation of a PIP joint fracture-dislocation. We explore the surgical technique's efficacy, focusing on its ability to restore joint congruity, range of motion, and overall hand function. Through this case, we aim to contribute to the body of evidence supporting this innovative approach and discuss its place in the treatment algorithm for complex PIP joint injuries.

## Case presentation

A 23-year-old right-handed male presented to our clinic six weeks following a sports-related injury to his left index finger while trying to catch a cricket ball. He reported an initial swelling and pain post-injury but did not seek immediate medical attention. His medical history was unremarkable, with no previous hand injuries or chronic illnesses.

**Figure 1:**
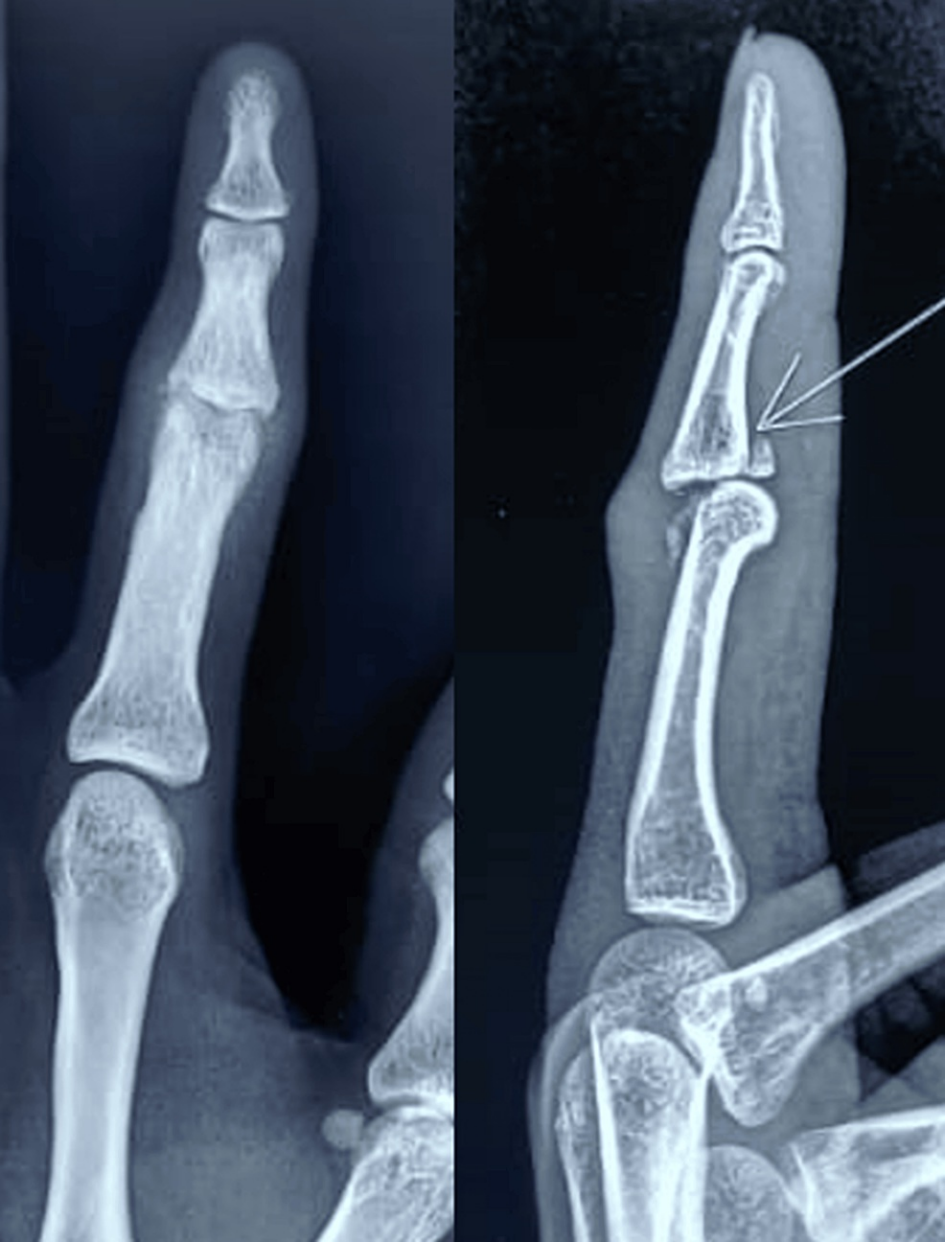
Standard radiographs confirmed a displaced intra-articular fracture of the base of the middle phalanx with subluxation of the PIP joint PIP, proximal interphalangeal

On examination, the patient exhibited notable swelling and tenderness over the left index finger's PIP joint. There was a significant reduction in the range of motion, with the patient unable to flex or extend the joint fully. A palpable step-off at the joint suggested a displaced fracture with subluxation of the joint. Neurovascular examination of the digit remained intact. Standard radiographs confirmed a displaced intra-articular fracture of the base of the middle phalanx with subluxation of the PIP joint, consistent with a complex fracture-dislocation (Figure 1).

**Figure 2:**
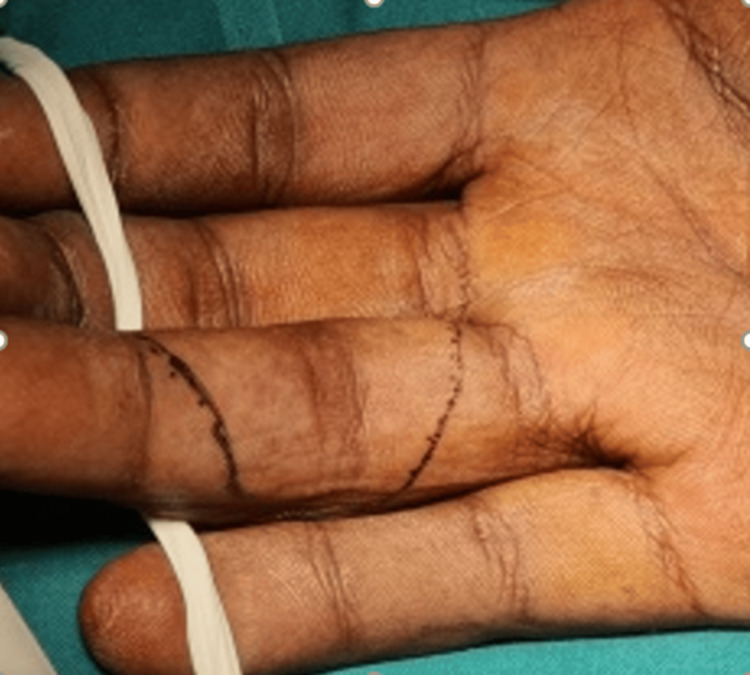
A volar approach with Bruner skin incision was used to expose the joint

Given the nature of the injury and the delay in presentation, a decision was made to proceed with hemi-hamate arthroplasty to restore the joint surface and function. Under regional anesthesia, a volar shotgun approach was used to expose the joint (Figure 2).

**Figure 3:**
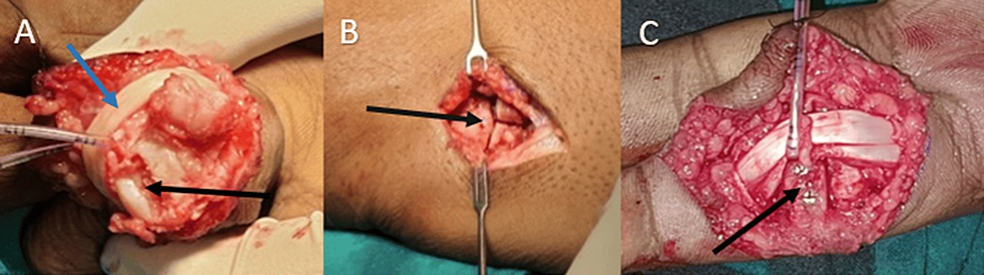
(A) Flexor tendons are retracted laterally (blue arrow) after incising the flexor tendon sheath and dividing the palmar plate distally and laterally. The finger is then hyperextended (shotgunned) to completely expose the fractured base of the middle phalanx (black arrow) and the head of the proximal phalanx. (B) A dorsal longitudinal incisional is taken over the carpometacarpal joint between the hamate and bases of the fourth and fifth metacarpals (black arrow). Hamate graft of appropriate size is harvested using an osteotome. (C) Graft was shaped and fixated onto the base of the middle phalanx using 1.3 mm cortical screws (black arrow).

The fractured pieces were removed, and an osteochondral graft was harvested from the patient's hamate. The graft was meticulously shaped and fixated onto the base of the middle phalanx using 1.3 mm cortical screws, effectively reconstructing the articular surface (Figure 3).

The joint was then realigned, and stability was tested through the range of motion. The wound was closed in layers, and a protective splint was applied.

**Figure 4:**
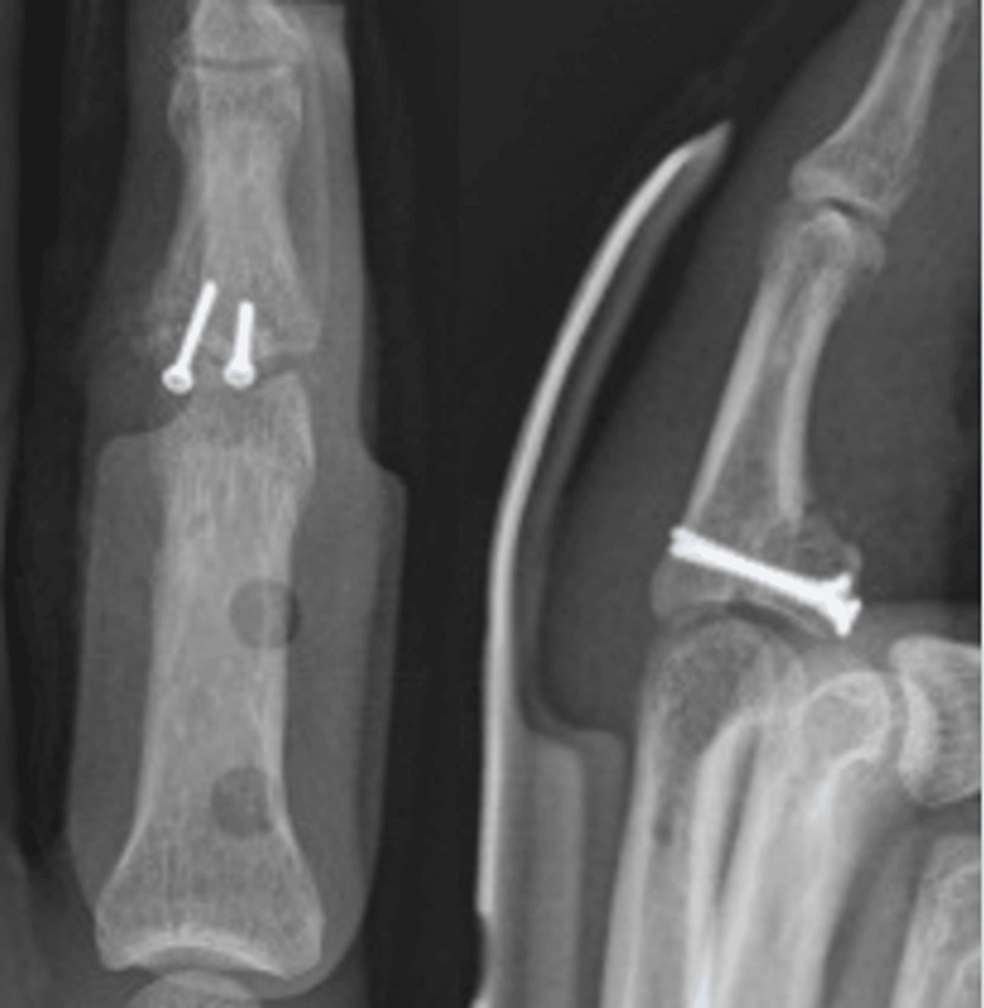
Patient's index finger was immobilized for one week post-surgery

The patient’s index finger was immobilized for one week post-surgery before initiating a structured hand therapy program focusing on gentle active and passive motion exercises (Figure 4).

Pain management and anti-inflammatory medications were prescribed to manage the initial postoperative discomfort.

**Figure 5:**
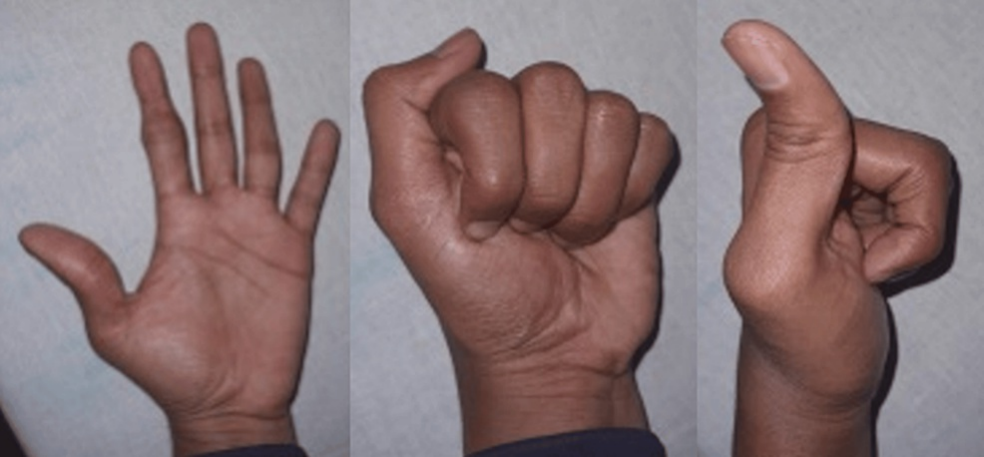
At the end of five months, the patient achieved 90 degrees of flexion and full extension with minimal discomfort

The patient was followed up at regular intervals over five months. At each visit, clinical and radiographic assessments were performed. The patient demonstrated progressive improvement in pain and range of motion at the PIP joint. By the end of five months, he achieved 90 degrees of flexion and full extension with minimal discomfort (Figure 5).

**Figure 6:**
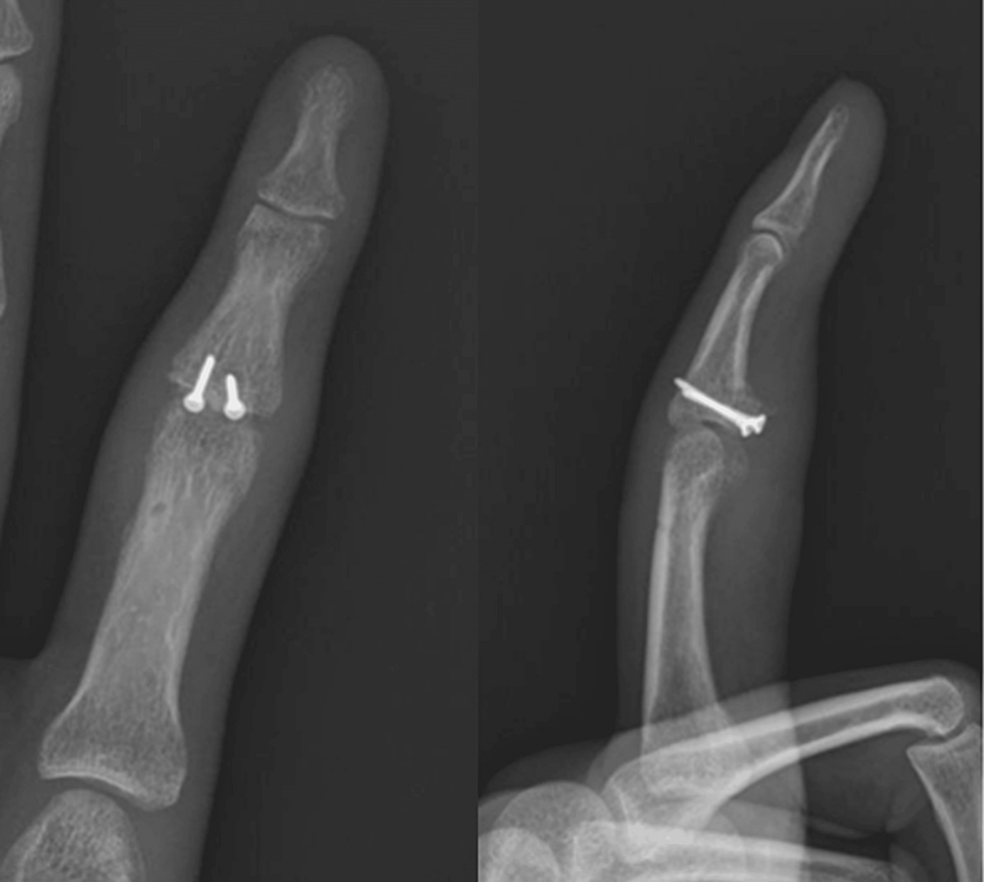
Five months postoperative radiographs showing good alignment and integration of the graft, with no signs of resorption or joint degeneration

Radiographs showed good alignment and integration of the graft, with no signs of resorption or joint degeneration (Figure 6).

The patient expressed satisfaction with the functional and cosmetic outcomes, reporting a significant improvement in his ability to perform daily activities and a return to recreational sports.

## Discussion

This case of a delayed presentation of PIP joint fracture-dislocation treated with hemi-hamate arthroplasty highlights several critical aspects of hand injury management. The successful outcome in this patient underscores the potential of this surgical technique in restoring joint function and anatomy, especially in complex cases where traditional methods may not be viable.

Hemi-hamate arthroplasty has been shown to provide favorable outcomes in terms of anatomical restoration and functional recovery. Technical difficulty of ORIF is increased by articular surface fracture comminution [5,6]. Compared to other reconstructive options, this method offers a more anatomical and congruent joint surface reconstruction, which is critical for the range of motion and long-term joint health. Our case supports these findings, demonstrating significant functional improvement post-surgery.

The technical aspects of hemi-hamate arthroplasty are crucial for its success [4]. Precise graft shaping and placement are imperative to ensure joint stability and congruity. Our experience aligns with the literature, suggesting that meticulous surgical technique significantly impacts patient outcomes.

Managing PIP joint injuries with delayed presentation poses unique challenges. Over time, joint stiffness, tissue contracture, and cartilage damage can complicate reconstruction and recovery [1]. This case adds to the growing evidence that hemi-hamate arthroplasty can be effectively employed even in delayed scenarios, providing a viable solution when other methods might fall short.

While this case demonstrates excellent short-term outcomes, long-term follow-up is essential to understand the durability of hemi-hamate arthroplasty. Literature suggests good long-term function and joint preservation, but ongoing surveillance is recommended to monitor for potential late complications such as joint degeneration or graft resorption.

As the body of evidence grows, further studies are needed to optimize patient selection, refine surgical techniques, and better understand the long-term outcomes of this procedure. Comparative studies with larger cohorts are particularly valuable to establish hemi-hamate arthroplasty as a standard treatment for complex PIP joint injuries.

## Conclusions

This case report of hemi-hamate arthroplasty for a delayed presentation of a PIP joint fracture-dislocation has demonstrated a successful outcome, with the patient achieving a significant improvement in range of motion and pain reduction. The procedure provided a viable reconstructive option, restoring joint congruity and function in a complex scenario where other treatment options might be less effective.

The favorable outcome in this case aligns with the existing literature, supporting hemi-hamate arthroplasty as a reliable technique for anatomically challenging cases of PIP joint injuries, particularly those with a delayed presentation. It underscores the importance of considering this surgical option in cases where traditional methods might fail to restore joint anatomy and function adequately.

However, it is crucial to acknowledge that while the short-term results are promising, the long-term success of hemi-hamate arthroplasty requires further investigation. As with any surgical intervention, careful patient selection, meticulous surgical technique, and a structured rehabilitation program are critical to optimizing outcomes. Future research should focus on larger cohort studies to validate the long-term efficacy and durability of this procedure and to refine patient selection criteria and surgical techniques.

In conclusion, this case contributes to the growing body of evidence supporting hemi-hamate arthroplasty as a beneficial option for reconstructing the PIP joint in complex fracture-dislocations. It highlights the procedure's potential in restoring hand function and improving patient quality of life, encouraging further exploration and study in this field.
